# Is vigorous physical activity effective for preventing kidney stones?

**DOI:** 10.3389/fpubh.2025.1612347

**Published:** 2025-09-19

**Authors:** Yuanfu Wang, Qing Wei, Yue Dai, Sisi Chen, Bo Cheng

**Affiliations:** ^1^Department of Urology, The Affiliated Hospital of Southwest Medical University, Southwest Medical University, Luzhou, Sichuan, China; ^2^Department of Biochemistry and Molecular Biology, School of Basic Medical Sciences, Southwest Medical University, Luzhou, Sichuan, China

**Keywords:** daily vigorous physical activity time, kidney stones, National Health and Nutrition Examination Survey (NHANES), moisture, logistic regression

## Abstract

**Background:**

With a high rate of occurrence and recurrence, kidney stones represent a common urological issue that poses a substantial burden on public health infrastructures globally. While prior research has linked poor diet and lifestyle to a heightened susceptibility to kidney stones, the impact of daily vigorous physical activity (VPA) duration on kidney stone incidence remains under-investigated.

**Materials and methods:**

Utilizing data from the NHANES database covering the years 2007 to 2020, this study undertakes a large-scale cross-sectional analysis of adults with full records of daily VPA and kidney stone history. Daily VPA time was calculated by summing the VPA duration (in minutes) from typical work and recreational activities. To analyze the association between VPA time and kidney stone prevalence, logistic regression was used, with a focus on potential non-linear relationships. A piecewise linear model estimated threshold effects, accompanied by subgroup and interaction analyses.

**Results:**

Of the 12,128 participants in this analysis, 1,021 (8.41%) had previously experienced kidney stones. Findings indicated a positive correlation between the duration of daily VPA and kidney stone prevalence. In the analysis of VPA time divided into quartiles, the highest quartile exhibited a 1.49-fold increase in kidney stone prevalence vs. the lowest quartile (OR = 1.49, 95% CI: 1.21–1.83, *P* for trend < 0.001). A smoothing curve fit showed a significant non-linear relationship between VPA time and kidney stones prevalence (*P* for non-linearity = 0.0007). Piecewise linear regression indicated a VPA threshold of 240 min, after which kidney stone prevalence increased by 0.3% for each additional minute of daily VPA (OR = 1.003, 95% CI: 1.000–1.006), up to 360 min, at which point the prevalence plateaued.

**Conclusion:**

This study suggests that VPA is associated with an increased risk of kidney stones, as longer daily VPA duration corresponds to a higher prevalence of kidney stones. This increase in prevalence may be related to the higher urine specific gravity caused by prolonged VPA. To strengthen these findings, future prospective cohort studies are recommended.

## Introduction

Globally, kidney stones are a common urological issue, characterized by high rates of incidence and recurrence, which heavily impact public health systems in many nations (1). About 10% of men and 7% of women in the U.S. will encounter kidney stones over the course of their lifetime (2). In China, kidney stone prevalence is also rising, with rates of 7%−9% observed in economically developed eastern regions (3). Although the direct mortality rate of kidney stones is low, severe complications such as urinary tract infections and kidney damage can be fatal (4). Furthermore, the recurrent nature of kidney stones imposes substantial medical costs, including diagnostics, surgical treatments, and long-term medication (5, 6). Evidence suggests that annual U.S. healthcare expenses for kidney stones top $2 billion, creating a notable strain on healthcare systems (7). As a result, a clear understanding of the causes and contributors to kidney stones is critical for preventing the disease.

Moderate exercise has a protective effect against kidney stones (8). However, since the physiological pathways triggered by VPA and moderate exercise are significantly different (9), the impact on kidney stones cannot be simply understood as the effect of exercise on stones. Vigorous physical activity (VPA) is typically defined as physical activity that significantly elevates heart and respiratory rates, often reaching 70%−85% or more of maximum heart rate. VPA is widely endorsed for its various health benefits (10). Research shows that regular VPA can improve cardiovascular function, increase cardiac output, and enhance circulation, helping reduce the risks of heart disease, hypertension, and stroke (11). Additionally, VPA significantly boosts energy expenditure, promoting fat burning, which supports body fat reduction and healthy weight maintenance (12). VPA has also been associated with improved insulin sensitivity, reducing the risk of insulin resistance (13). VPA can further alleviate symptoms of depression and anxiety, enhancing mental health (14), VPA also contributes to enhancing muscle strength and bone health (15). Despite these benefits, VPA carries certain potential drawbacks and risks. For individuals lacking conditioning, VPA may cause significant increases in heart rate and blood pressure, potentially leading to arrhythmia or even sudden cardiac arrest (16). Overloading the body with VPA, without proper warm-up or recovery, may result in common injuries, such as muscle strains, sprains, and joint pain (17, 18). Although VPA helps reduce stress and anxiety, excessive VPA may increase psychological stress (19). Thus, it is important to understand how VPA affects specific diseases to better guide exercise recommendations.

A considerable amount of research has explored the link between physical activity and kidney stones. Many studies have indicated that physical activity may protect against kidney stones (8, 20–22), while a sedentary lifestyle has been identified as a risk factor (23). Given the dual effects of VPA, focusing solely on whether physical activity affects kidney stones may be insufficient. Previous studies have not directly investigated the possible influence of VPA on the formation of urinary stones. Considering this, the study aims to evaluate the relationship of daily VPA time to kidney stones prevalence in U.S. adults, with data from NHANES.

## Methods

### Study population

This study sourced data from NHANES, a series of national surveys by the U.S. NCHS that aims to assess the health of U.S. citizens. NHANES employs a complex, multistage sampling approach (sampling counties, segments, households, and individuals), ensuring the U.S. population is well-represented. The NCHS Ethics Review Board approved NHANES, with informed consent obtained from all participants.

**Figure 1:**
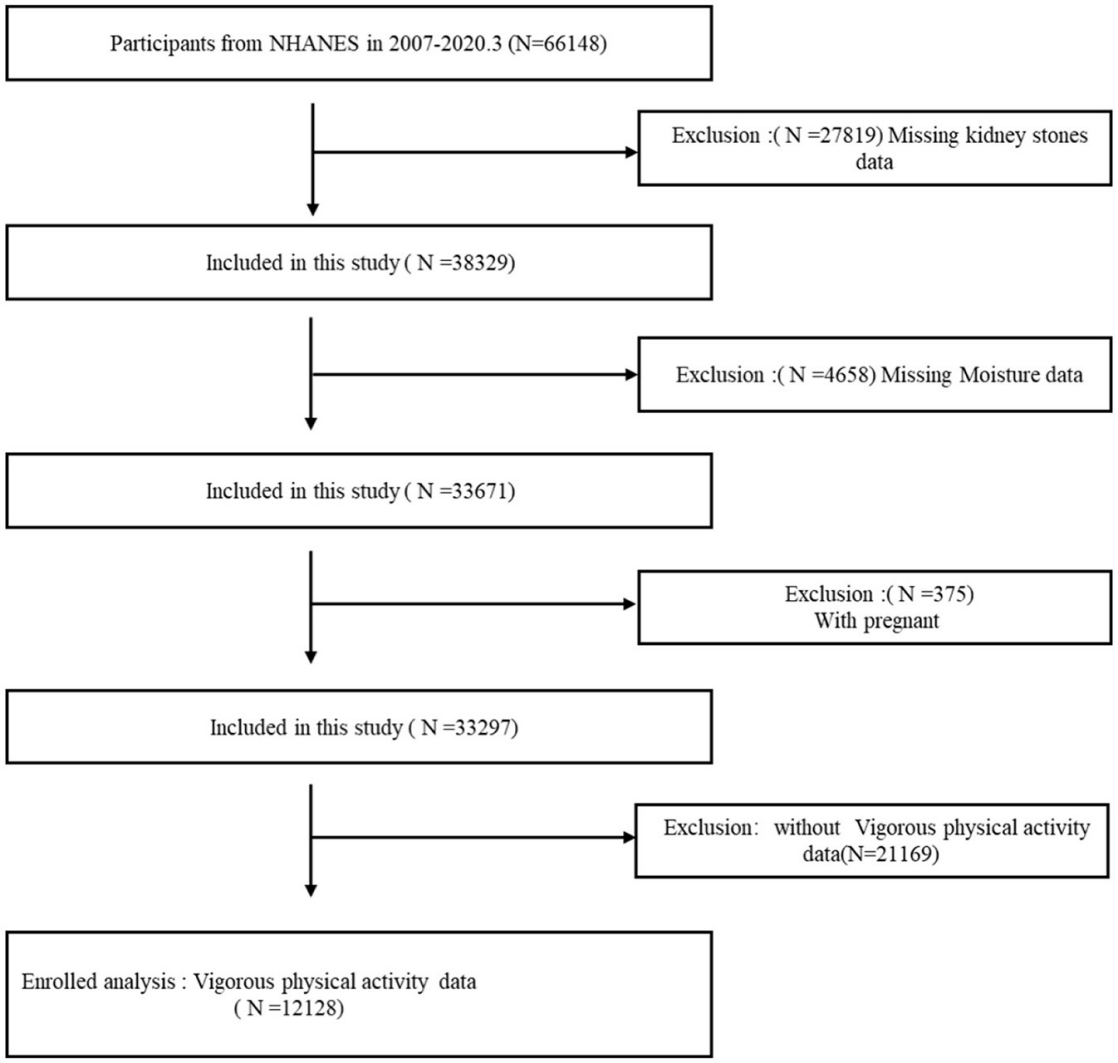
Flowchart depicting participant selection in the study. NHANES, National Health and Nutrition Examination Survey.

All study methods strictly adhered to relevant guidelines and regulations. For this specific study, we collected data from 66,148 participants across six consecutive NHANES cycles (2007–2020.03). Specific exclusion criteria were applied: (1) participants lacking kidney stone outcome data (*n* = 27,819); (2) individuals missing moisture or VPA data (*n* = 25,827); and (3) pregnant participants (*n* = 375). After careful data screening, 12,128 participants were selected for further analysis, with the participant selection process illustrated in Figure 1.

### Definition of kidney stones and VPA

The KIQ026 question in the Kidney Conditions-Urology survey was used to determine the presence of kidney stones in the questionnaire data. Those who affirmed having kidney stones by answering “yes” to the question, “Do you have kidney stones?” were defined as having a history of the condition.

In NHANES, participants answered a questionnaire on physical activity, based on the Global Physical Activity Questionnaire (GPAQ) (24), which recorded details about the type, frequency, and duration of their activities in the past 30 days. VPA was defined by responses to the Questionnaire, which collected data on the duration of vigorous physical activities lasting 10 min or more, including both work and recreational activities that elevate heart rate or breathing. METs (metabolic equivalents) were used to quantify energy expenditure, with 1 MET representing 3.5 ml O_2_·kg^−1^·min^−1^. Both vigorous work and recreational activities were assigned 8.0 METs, and the daily VPA time was determined by summing the durations of these activities.

### Covariates

Based on previous literature and biological considerations, we included a wide range of covariates known to influence kidney stones' outcomes. The covariates included gender, age, race/ethnicity, education level, poverty index ratio (PIR), and body mass index (BMI) hypertension, diabetes, smoking status, alcohol intake (yes/no), moisture, blood urea nitrogen, creatinine, uric acid, eGFR, total calcium, moderate physical activity time, and sedentary time. Participants were categorized by BMI: < 24 kg/m^2^ as normal weight, 24–28 kg/m^2^ as overweight, and ≥28 kg/m^2^ as obese. Smoking status was obtained through household interviews and classified as “never,” “current,” “sometimes,” or “past.” The moisture refers to the sum of water consumed directly as drinking water and the water content in food and non-water beverages (25), calculated as the average of values from the first and second dietary interviews. The blood urea nitrogen, creatinine, uric acid, and total calcium levels are derived from the standard biochemical overview section of laboratory data. The estimated glomerular filtration rate (eGFR) is calculated using the following equation (26):

$$\begin{array}{l} \text{eGFR}=141\times \text{min}{\left(\text{Scr}/\alpha ,1\right)}^{\text{β}}\times \text{max}{\left(\text{Scr}/\alpha ,1\right)}^{-1.209}\times 0.993^{\text{age}}\times \gamma \\ \;\;\times 1.159\left(\text{if black}\right). \end{array}$$

Where:

- For males: α = 0.9, β = −0.411, γ = 1
- For females: α = 0.7, β = −0.329, γ = 1.018

Moderate physical activity time equaled the sum of moderate work time, moderate recreational activity time, and walking or cycling time. Detailed measurements of these variables can be found at www.cdc.gov/nchs/nhanes/.

### Statistical analysis

Statistical analyses were carried out in accordance with CDC guidelines, applying NHANES sampling weights and considering complex, multistage cluster design. Continuous variables were reported as means with standard deviations (SD), and categorical variables as proportions. Weighted *t*-tests and chi-square tests were used to evaluate differences between participants with and without kidney stones. To evaluate how daily VPA time relates to kidney stone prevalence, we used multivariable logistic regression models (Models 1 and 2), to calculate odds ratios (ORs) and 95% confidence intervals (CIs). In Model 1, adjustments were made for gender, age, and ethnicity. Model 2 was adjusted for gender, age, ethnicity, education level, PIR, BMI, hypertension, diabetes, smoking, alcohol intake, moisture, blood urea nitrogen, creatinine, uric acid, eGFR, total calcium, moderate physical activity time, and sedentary time. The non-linear relationship between daily VPA time and kidney stone prevalence was also assessed using smooth curve fitting, and a two-segment linear regression model was applied to estimate the inflection point. Subgroup analyses were performed, with gender, age, BMI, hypertension, and diabetes treated as potential effect modifiers. Likelihood ratio tests were used to introduce and evaluate interaction terms to quantify heterogeneity. Missing values were imputed using the median for continuous variables or the mode for categorical variables. R (version 4.4.1) and Empower software (www.empowerstats.com; X&Y Solutions, Inc., Boston,) were applied to perform statistical analyses, we considered a *P*-value of less than 0.05 to be statistically significant.

## Results

### Baseline characteristics of participants

A total of 12,128 participants were included in this study, of whom 1,021 (8.41%) had a history of kidney stones. After weighting, 67.52% of the included participants were male and 32.48% were female. Table 1 displays the weighted distribution of covariates among individuals with and without kidney stone history. Age (*P* < 0.001), gender (*P* = 0.004), race/ethnicity (*P* < 0.001), smoking status (*P* < 0.001), hypertension (*P* < 0.001), diabetes (*P* < 0.001), BMI (*P* < 0.001), blood urea nitrogen (*P* < 0.001), creatinine (*P* = 0.001), uric acid (*P* = 0.042), eGFR (*P* < 0.001), total calcium (*P* = 0.002), and moderate physical activity (*P* = 0.0395) were all significantly associated with the presence of kidney stones. Kidney stone sufferers were typically older, male, non-Hispanic White, and had higher incidences of smoking, hypertension, diabetes, higher BMI, poor kidney function, and more time on moderate physical activity. However, kidney stone status was not significantly associated with education level, PIR, alcohol intake, moisture, or sedentary time (all *P* > 0.05). Table 2 describes differences in exposure variables between participants with and without kidney stones; those with a history of kidney stones had a higher mean VPA time (166.42 ± 147.60 min) than those without (147.23 ± 139.00 min, *P* < 0.001). Quartile comparisons of VPA revealed that those who had kidney stones typically spent more time on daily VPA.

**Table 1:** Baseline of weighted characteristics of participants with and without history of kidney stone: NHANES survey 2007–2020.03.

**Characteristics**	**Participants without kidney stone**	**Participants with kidney stone**	**Standardize diff**.	***P*-value**
*N*	11,107	1,021		
Age (mean ± SD)	41.29 ± 14.90	48.55 ± 14.63	0.51 (0.45, 0.58)	< 0.001
**Sex**, ***n*** **(weighted %)**			0.09 (0.03, 0.16)	0.004
Male	6,882 60.61%)	679 (67.52%)		
Female	4,225 (39.39%)	342 (32.48%)		
**Hypertension**, ***n*** **(weighted %)**			0.37 (0.30, 0.43)	< 0.001
No	8,322 (78.22%)	591 (61.96%)		
Yes	2,785 (21.78%)	430 (38.04%)		
**Diabetes**, ***n*** **(weighted %)**			0.30 (0.23, 0.36)	< 0.001
No	10,013 (93.38%)	813 (84.20%)		
Yes	1,094 (6.62%)	208(15.80%)		
**BMI**, ***n*** **(weighted %)**			0.29 (0.23, 0.36)	< 0.001
≤ 24	2,894 (27.96%)	161 (15.41%)		
>24, ≤ 28	3,100 (29.81%)	262 (27.37%)		
>28	5,060 (42.22%)	596 (57.22%)		
**Moisture (gm)**, ***n*** **(weighted %)**			0.06 (-0.00, 0.13)	0.348
≤ 1,000	161 (0.99%)	12 (0.79%)		
>1,000, ≤ 3,000	6,053 (49.46%)	563 (50.16%)		
>3,000, ≤ 5,000	3,911 (39.33%)	371 (41.67%)		
>5,000	982 (10.21%)	75 (7.37%)		
**Uric acid (mg/dL)**, ***n*** **(weighted %)**			0.09 (0.03, 0.16)	0.042
0.8–4.5	2,746 (24.98%)	234 (22.17%)		
4.6–5.4	2,666 (24.95%)	235 (23.74%)		
5.49–6.2	2,789 (24.35%)	243 (25.04%)		
6.3–13.3	2,906 (25.73%)	309 (29.05%)		
**eGFR [mL/(min. 1.73 m**^2^**)]**, ***n*** **(weighted %)**			0.25 (0.18, 0.31)	< 0.001
45.05–83.56	2,674 (24.88%)	356 (35.54%)		
83.57–103.99	2,789 (27.45%)	245 (26.34%)		
104–120.35	2,815 (24.84%)	217 (21.72%)		
120.36–206.35	2,829 (22.83%)	203 (16.41%)		

**Table 2:** Quartile of vigorous physical activity between stones and non-stone group.

**Characteristics**		**Participants without kidney stone**	**Participants with kidney stone**	**Standardize diff**.	***P*-value**
*N* (%)		11,107 (91.58%)	1,021 (8.49%)		
VPA (min, mean + SD)		147.23 ± 139.00	166.42 ± 147.60	0.13 (0.07, 0.20)	< 0.001
VPA quartile (min)		*N* (%)	*N* (%)	0.15 (0.09, 0.22)	< 0.001
Q1	10–55 (min)	2,726 (24.54%)	220 (21.55%)	–	–
Q2	60–102 (min)	2,869 (25.83%)	232 (22.72%)	–	–
Q3	105–230 (min)	2,765 (24.89%)	250 (24.49%)	–	–
Q4	240–480 (min)	2,747 (24.73%)	319 (31.24%)	–	–

### Logistic regression analysis and smooth curve fitting of kidney stone prevalence

**Figure 2:**
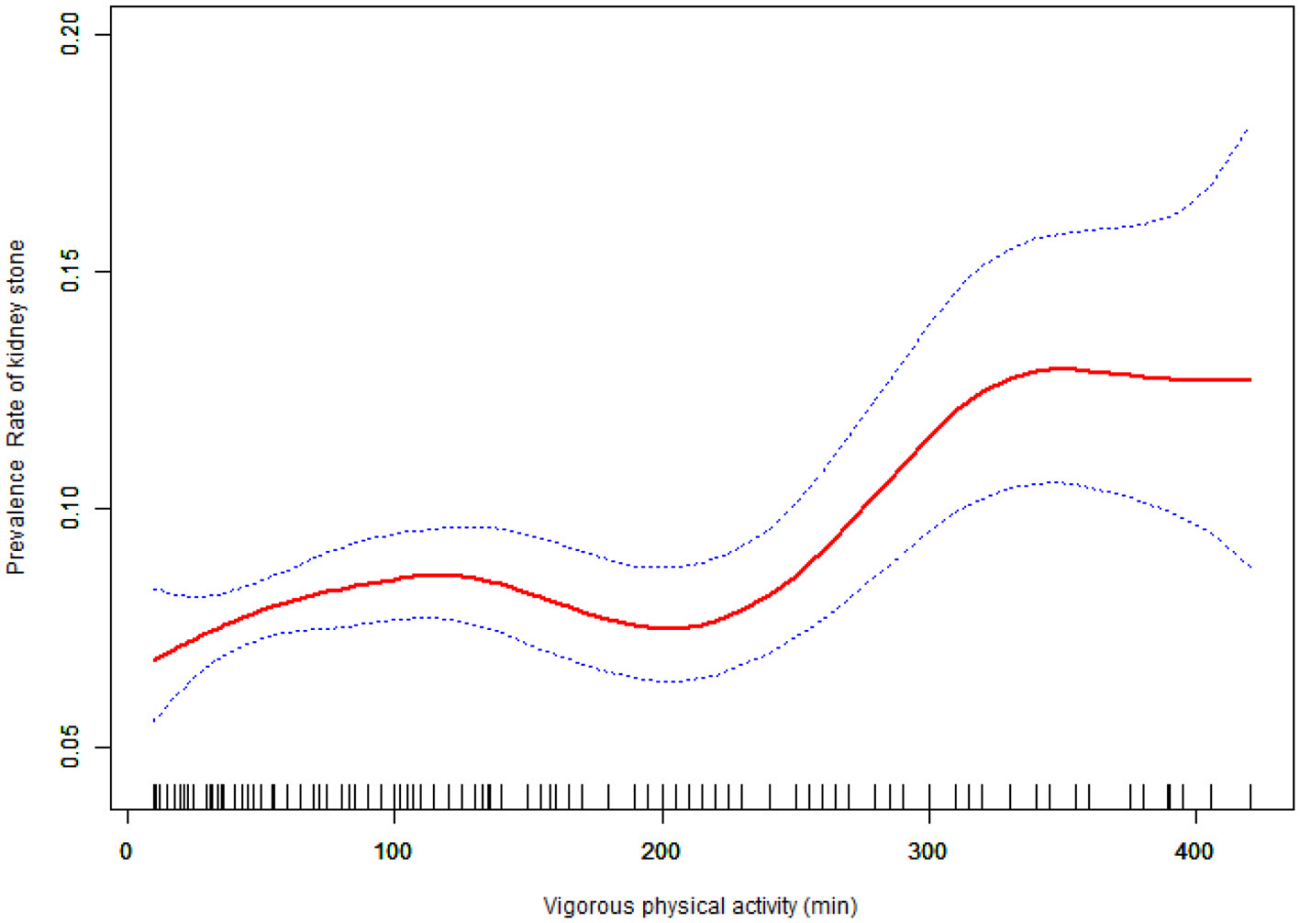
The curve of the association between Vigorous physical activity and prevalence rate of kidney stones among study participants in fully adjusted model.

Table 3 shows the association between VPA and kidney stone prevalence. Results indicate that an increase in daily VPA time corresponded with a higher prevalence of kidney stones. When daily VPA time was divided into quartiles, multivariable logistic analysis of the fully adjusted model indicated that VPA was a risk factor for kidney stones; compared to the lowest quartile, kidney stone prevalence increased as daily VPA time rose. Participants in the top quartile of VPA showed a kidney stone prevalence that was 1.49 times greater than those in the bottom quartile (OR = 1.49, 95% CI: 1.21–1.83; *P* for trend < 0.001). To further clarify the association between daily VPA time and kidney stone prevalence, we used smooth curve fitting (Figure 2). After excluding extreme values (daily VPA time < 5% and >95%), smooth curve fitting showed a significant non-linear trend between VPA and kidney stone prevalence (*P* for non-linear trend = 0.0007).

**Table 3:** OR (95 % CI) of prevalence rate of kidney stones by quartile of various physical activity.

**Characteristics**	**Prevalence rate of kidney stones**			
**Model**	**Adjust I**		**Adjust II**	
**VPA (min)**	**OR (95%CI)**	***P*** **-value**	**OR (95%CI)**	***P*** **-value**
Q1	1.0		1.0	
Q2	1.05 (0.86, 1.28)	0.624	1.11 (0.91, 1.36)	0.645
Q3	1.13 (0.93, 1.37)	0.218	1.16 (0.95, 1.43)	0.174
Q4	1.48 (1.22, 1.78)	< 0.001	1.49 (1.21, 1.83)	< 0.001
*P*-value for VPA group trend	*p* < 0.001		*p* < 0.001	

Results in the table: OR (95%CI) *P*-value.

Adjust I model adjust for: Age; Sex, Ethnicity. Adjust II model adjust for: Age; Sex; Ethnicity; Edu; PIR; Smoke; Alcohol drinking; Hypertension; Diabetes; BMI, Moisture intake., Moderate physical activity, Sedentary activity, blood urea nitrogen, creatinine, uric acid, eGFR and total calcium.

As daily VPA time increased, kidney stone prevalence rose gradually until reaching a threshold where prevalence sharply increased. Using a two-segment linear regression model, we calculated a VPA threshold (K) of 240 min (Table 4). With increasing duration of daily vigorous physical activity (VPA), the prevalence of kidney stones gradually rises, followed by a sharp increase after reaching the threshold of 240 min. Before the threshold ( ≤ 240 min), VPA duration was not significantly associated with kidney stone prevalence (OR = 1.001, 95% CI: 1.000–1.002); however, beyond this threshold, each additional minute of VPA was significantly associated with a higher prevalence (OR = 1.003, 95% CI: 1.000–1.006), and this trend plateaued after approximately 360 min.

**Table 4:** Threshold effect analysis of the daily vigorous physical activity time and prevalence rate of kidney stones.

**Outcome:**	**Prevalence rate of kidney stones**	
**Model**	**OR(95%CI)**	***P*** **-value**
Fitting by standard linear model	1.002 (1.001, 1.002)	< 0.001
**Fitting by two-piecewise linear model**		
Breakpoint (K)	240	
OR1 (VPA < 240)	1.001 (1.000, 1.002)	0.279
OR2 (VPA > 240)	1.003 (1.001, 1.006)	0.001
OR2/OR1	1.003 (1.000, 1.006)	0.045
Logarithmic likelihood ratio test *P*-value	0.046	

Adjust for: Age, Sex, Ethnicity, Edu, PIR; Smoke, Alcohol drinking, Hypertension, Diabetes, BMI, Moisture intake, Moderate physical activity, Sedentary activity, blood urea nitrogen, creatinine, uric acid, eGFR and total calcium.

### Subgroup analysis

**Figure 3:**
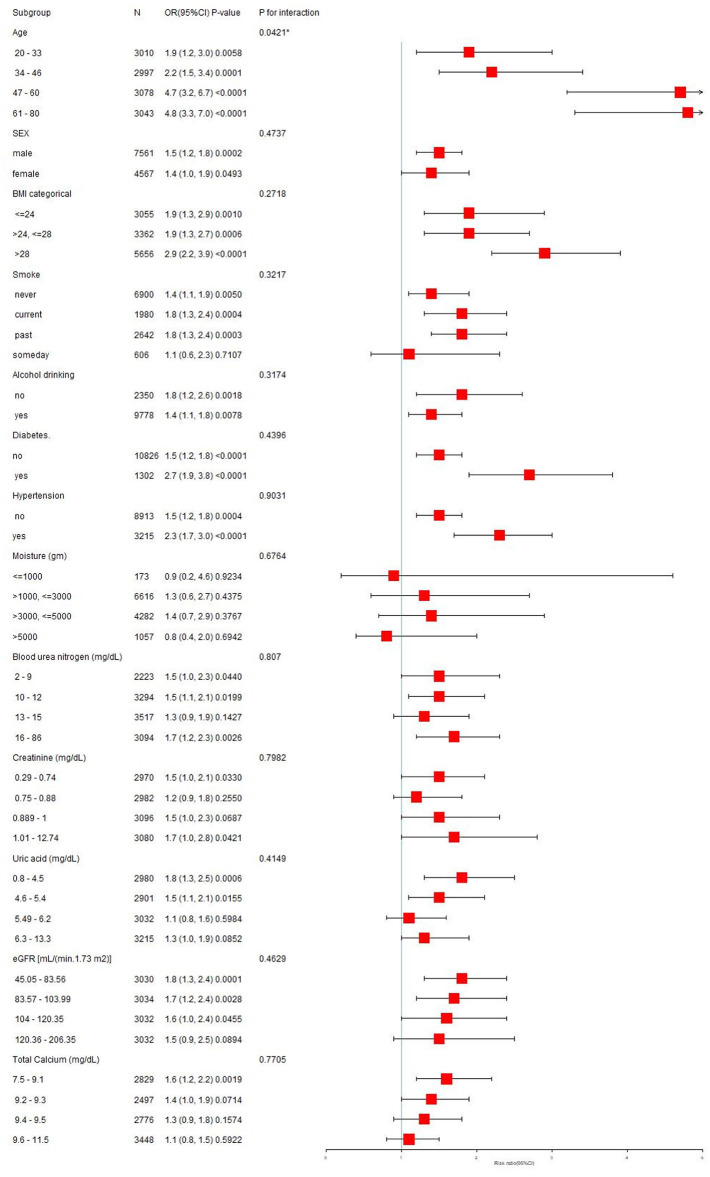
Subgroup analysis on the association of vigorous physical activity and the prevalence of kidney stones.

We divided daily VPA time into two segments based on the inflection point, with the first segment as the reference group, to conduct subgroup analyses examining the stability of the relationship between daily VPA time and kidney stone prevalence across different population subgroups. As illustrated in Figure 3, nearly all subgroup stratifications, including age, gender, BMI, alcohol intake, smoking, hypertension, blood urea nitrogen, creatinine, uric acid, eGFR, and total calcium, and diabetes status, did not significantly affect the positive correlation between daily VPA time and kidney stone prevalence. Interestingly, in the subgroup stratified by moisture, the significant positive correlation between daily VPA time and kidney stone prevalence disappeared (*P* > 0.05). Interaction tests showed that only age had a significant impact on the relationship between daily VPA time and kidney stone prevalence (*P* = 0.00421), this positive correlation was not significantly affected by gender, BMI, drinking, smoking, hypertension, diabetes, blood urea nitrogen, creatinine, uric acid, eGFR, total calcium, or moisture intake (all interaction terms, *P* > 0.05).

## Discussion

In this observational study, we observed a significant association between prolonged vigorous physical activity (VPA) duration and increased kidney stone prevalence after adjusting for confounders. Specifically, when daily VPA exceeded 240 min, each additional minute was linked to a 0.3% rise in prevalence, plateauing near 360 min. This pattern corresponded with increased urine specific gravity during extended VPA, suggesting dehydration may contribute to risk. These findings highlight the importance of targeted moisture strategies for high-exposure groups (e.g., athletes): clinicians should advise proactive moisture, before, during, and after prolonged VPA to mitigate urine concentration. Mechanistic confirmation requires further research: we propose longitudinal cohorts tracking VPA exposure against dynamic urine composition changes (calcium, citrate, pH), followed by randomized controlled trials (RCTs) testing moisture interventions on stone incidence in high-risk populations. While moisture remains, a practical precaution based on our data, definitive causal pathways warrant verification through these studies.

The association between physical activity and kidney stones has been a topic of much discussion. Liu et al., using data from the UK Biobank, found that physical activity was negatively associated with kidney stone disease (KSD) risk, irrespective of genetic predisposition (27). A non-linear relationship between physical activity and KSD was observed in The study by Feng et al. found that KSD prevalence decreased as physical activity rose, before leveling out at a certain threshold (20). Using NHANES data, Li et al. showed that in individuals who did not engage in vigorous recreational activity, increased sedentary time was associated with a higher prevalence of kidney stones (23). While some studies suggest physical activity protects against KSD, others have found no meaningful correlation. A cohort study of 215,133 participants observed no evident link between physical activity and KSD after accounting for various confounding factors (22). Similarly, Patrick et al.'s systematic review, which included 17,511 patients, found inconclusive evidence on the relationship of physical activity to KSD (28). In contrast, our study found that prolonged VPA was not protective against kidney stone.

It was associated with an increased risk. Li et al.'s research also suggested that after reaching a certain intensity and duration of exercise, kidney stone prevalence does not continue to decrease, consistent with our findings. Our study addresses a research gap by specifically examining the impact of vigorous physical activity on kidney stone prevalence.

In our study, vigorous activity appeared as a risk factor for kidney stones, likely due to multiple mechanisms. First, exercise-induced sweating and fluid loss may cause dehydration, leading to urine concentration and supersaturation of stone-forming substances, calcium oxalate and uric acid, for instance, may then deposit in the kidneys (29–31). Additionally, electrolyte imbalances from sweat loss could disrupt calcium metabolism, raising calcium ion concentration in urine and increasing the risk of calcium oxalate stones (31). Acidic metabolic by-products that accumulate during intense exercise may further acidify urine, promote the deposition of uric acid and calcium oxalate and thereby increasing kidney stone risk (32). Furthermore, vigorous exercise accelerates metabolism, increasing uric acid production, especially under dehydrated conditions that favor urate crystal formation (30). Based on these observations, we hypothesized that VPA may lead to urine concentration, with stone-forming substances reaching supersaturation and depositing in the kidneys, thus elevating kidney stone risk. To explore this hypothesis, we analyzed the relationship between daily VPA time and urine specific gravity. As depicted in Supplementary Table S1 and Supplementary Figure S1, after adjusting for various confounders, we found that vigorous exercise was associated with an increase in urine specific gravity (β = 0.07, 95% CI from 0.002 to 0.12, *P* = 0.00495), especially when VPA time exceeded 170 min, aligning with the K-value calculated using a two-segment linear regression model. Notably, when daily VPA time exceeded 360 min, the prevalence of kidney stones no longer increased, investigating the underlying mechanisms may be valuable. In the initial stages of exercise, sweating induces dehydration, electrolyte loss, elevated uric acid levels, and urine acidification, all of which collectively elevate the risk of kidney stone formation; however, as exercise continues (e.g., reaching 360 min), the body initiates a series of adaptive responses to mitigate this risk. These include: (1) activation of AMPK to inhibit xanthine oxidase activity, thereby reducing uric acid production; (2) enhanced activity of antioxidant enzymes such as superoxide dismutase (SOD) (33) and glutathione peroxidase (GPx), alleviating renal tubular damage; (3) restoration of urine dilution through rehydration and the antidiuretic hormone (ADH) escape mechanism; and (4) increased urinary citrate excretion, promoting calcium chelation and inhibiting crystal formation (34). Together, these mechanisms facilitate rehydration, restore electrolyte balance, regulate uric acid metabolism, and enhance acid-base buffering, ultimately enabling the body to progressively reduce the likelihood of kidney stone formation during prolonged exercise (35).

There are several strengths in our study. First, it is based on NHANES data, which is a nationally representative dataset obtained through standardized protocols. We performed all analyses using appropriate NHANES sampling weights, ensuring our findings reflect the broader population. We also controlled for a variety of confounding factors to ensure the robustness of our conclusions, and the large sample size allowed for detailed subgroup analyses. Moreover, based on available information, this study assesses the effect of daily VPA on kidney stone risk, clearly defining the type of exercise. Our results offer valuable insights for those in physically demanding occupations or recreational activities, with implications for kidney stone management. Despite the insights provided by this study, there are limitations. Primarily, because it is based on the NHANES database, we cannot establish causality for daily VPA time and kidney stone prevalence. Additionally, as NHANES data represents only the U.S. population, the generalizability of our findings may be limited. The self-reported nature of both the exposure and outcome variables introduces potential for recall and self-report biases, which could lead to the omission of asymptomatic kidney stone cases. We acknowledge the absence of important confounders [e.g., diet (36), dietary supplement intake (37), oxalate intake, genetics (38), occupation (39), climate (40)] due to data limitations. Reverse causality is also a possibility. These are now explicitly listed as limitations, and we propose future cohort studies and Mendelian randomization to further clarify causal relationships. Additionally, variations in moisture reporting between participants with and without kidney stones could introduce reporting bias. Specifically, those with a history of kidney stones may report higher moisture, which may account for the lack of a significant positive correlation between daily VPA time and kidney stone prevalence in our subgroup analysis of moisture. Although VPA itself can lead to short-term dehydration, which is a known theoretical risk factor for kidney stone formation, in real-world settings—particularly when individuals maintain adequate fluid regulation—the net effect of VPA on kidney stone risk tends to be neutral or even beneficial. This is primarily because sufficient water intake plays a decisive role in urine dilution, effectively mitigating the concentration of urine caused by intense physical activity (41). In populations with low or no fluid intake, the duration of VPA may be limited (since prolonged exercise without hydration can lead to severe consequences), and previous findings suggest that short-term vigorous activities—such as swimming—may even have protective effects (42). While it is true that VPA can lead to dehydration, the key factor is the individual's hydration habits during and after exercise. If exercisers—especially those who engage in regular, health-conscious physical activity—actively replenish fluids during and after workouts, they can effectively reverse the temporary urine concentration caused by exercise. Overall, adequate total moisture intake is one of the most effective strategies for preventing kidney stones (43).

## Conclusion

Our analysis demonstrates that, in this observational study, longer durations of VPA are linked to a higher prevalence of kidney stones after accounting for potential confounders. Specifically, we observed that kidney stone prevalence increased with rising daily VPA time. When daily VPA time exceeded 240 min, each additional minute of VPA was associated with a 0.3% increase in kidney stone prevalence, reaching a plateau around 360 min. This observed association may be related to the concurrent increase in urine specific gravity seen with prolonged VPA. Given these findings, maintaining adequate moisture during periods of VPA could be a prudent practical measure.

## Data Availability

Publicly available datasets were analyzed in this study. This data can be found here: https://wwwn.cdc.gov/Nchs/Nhanes/search/default.aspx.
