# Supplementary material for: Is vigorous physical activity effective for preventing kidney stones?
Source: Front Public Health. 2025 Sep 19;13:1612347. doi: 10.3389/fpubh.2025.1612347 (PMC12491037; doi:10.3389/fpubh.2025.1612347)
Supplement: Supplementary file 1 [file Table_1.docx]

| **Characteristics** | **1000*（Urine Specific Gravity - 1)** | | | | |
| --- | --- | --- | --- | --- | --- |
| **model** | **Adjust I** | | | **Adjust II** | |
| **VPA (min)** | **β (95%CI)** | | **P value** | **β (95%CI)** | **P value** |
| **Q1** | 1.0 | |  | 1.0 |  |
| **Q2** | 0.024 (-1.136, 1.183) | | 0.968 | 0.117 (-1.055,1.289) | 0.845 |
| **Q3** | 0.485 (-0.659, 1.628) | | 0.406 | 0.714 (-0.446,1.874) | 0.228 |
| **Q4** | 1.090 (-0.085, 2.265) | | 0.069 | 1.496 (0.299, 2.692) | 0.014 |
| **VPA(min) group trend** | 0.032 |  | | 0.005 | |

Adjust I model adjust for: Age, Sex, Ethnicity.
Adjust II model adjust for: Age , Sex ,Ethnicity , Edu, PIR ,Smoke , Alcohol drinking , Hypertension, Diabetes , BMI, Moisture intake, Moderate physical activity, Sedentary activity.
