# Supplementary figures and images for: Is vigorous physical activity effective for preventing kidney stones?
Source: Front Public Health. 2025 Sep 19;13:1612347. doi: 10.3389/fpubh.2025.1612347 (PMC12491037; doi:10.3389/fpubh.2025.1612347)

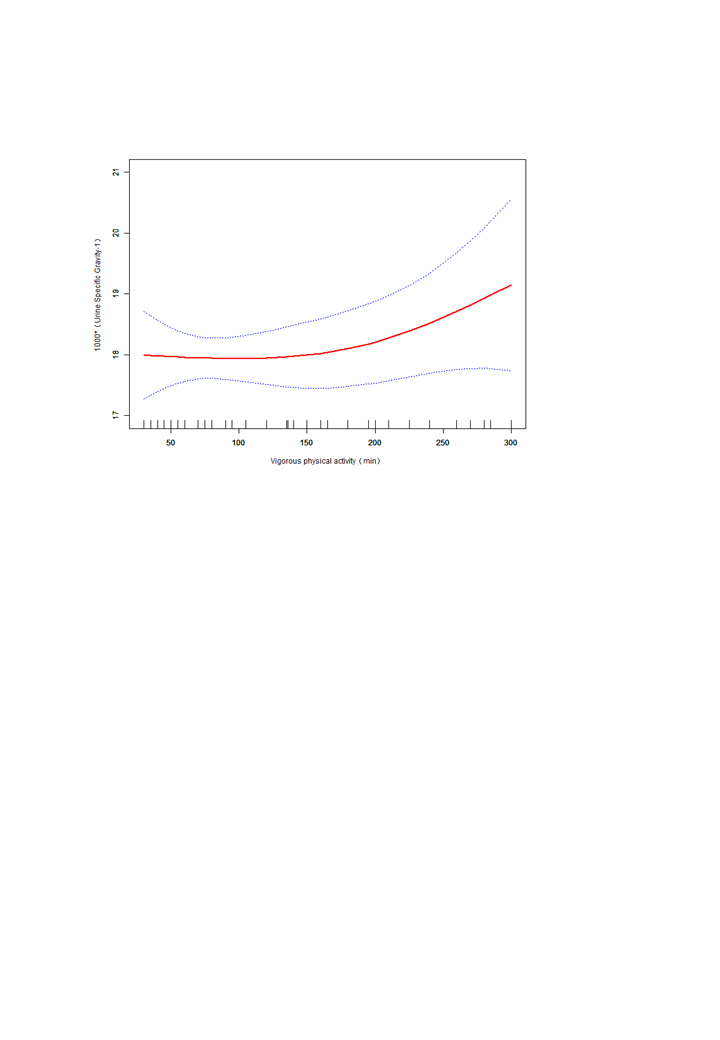

Supplement: Supplementary file 2 [file Image_1.tif]
